# Cebebro-Spinal Meningitis

**Published:** 1872-05-01

**Authors:** E. Fletcher Ingals

**Affiliations:** House Physician to Cook County Hospital


### CEREBRO-SPINAL MENINGITIS.

### MARKED SYMPTOMS TREATED WITH CANNABIS INDICA, IN LARGE DOSES. SPEEDY AND ALMOST PERFECT RECOVERY.

REPORTED BY E. FLETCHER INGALS, M.D.,

(*House Physician to Cook County Hospital.*)

M— C—; æt. 2 years. This case occurred in the practice of another physician (Dr. Chenoweth), who, after the first visit, narrated the case to me, and consulted me with regard to the treatment by cannabis indica. We decided upon large doses (for a child) of the tincture, which doses were to be rapidly increased, and the symptoms carefully watched, until a decided effect was obtained. Subsequently I saw the case; and since its recovery, the doctor has kindly furnished me with a few notes on its progress.

The case was first seen April 15th, at which time the friends stated that the child had been taken with violent vomiting the previous day. They further stated that a brother of the little girl, nine years of age, had died only a day or two previously, after a sickness of only a few hours. The only symptom which they remembered in his case, was that of vomiting. At this visit, the little girl wa found suffering from anorexia, constipation, great restlessness, and marked opisthotonos. The head and heels were drawn strongly back, so that the child rested upon them only, when the child was turned upon its back. If turned upon the abdomen, the head and heels were raised, the patient resting only on the trunk. The slightest movement of the child caused her to cry bitterly. Tinct. cannabis was prescribed. The dose contained ten drops, and was ordered to be repeated, with the addition of one drop every two hours, until twenty drops were taken at a dose.

For the constipation, leptandria and jalapa were ordered. Two days later (April 17th), pulse 150; active symptoms continuing.

April 18th. Pulse 149; resp. 30; temperature somewhat increased; tongue coated brownish white; and pupils contracted. Repeated the cathartic, and obtained a free movement during the day. The patient was then taking twenty drops of the cannabis every two hours.

April 19th. Pulse 140; resp. 36; skin cool.

April 20th. Pulse 128; resp. 26; skin cool; bowels regular. The patient had slept well during the preceding night; and the head was not so much retracted. The medicine was ordered to be continued. Two days later, the patient was much improved; the pulse was 108; the respirations 26; and the patient had a fair appetite.

The following day, the pulse and respirations were somewhat accelerated, but the general appearance of the patient was improved.

The following day (April 24th), the symptoms denoted improvement. The head was less retracted; the child was reported to have slept well, and began to be interested in its toys; however, constipation still existed; and the child was very irritable. A cathartic was prescribed, and the cannabis ordered in doses of ʒ ss. of the tincture every three hours.

April 26th. Resp. 30; pulse 120; digestive organs in a healthy condition; and opisthotonos scarcely perceptible. The medicine was continued in the same doses.

April 29th. Fifteen days after the inception of the disease, the child was apparently perfectly well, with the exception of a slight uncertainty in the gait.

